# Cooperative RNA Polymerase Molecules Behavior on a Stochastic Sequence-Dependent Model for Transcription Elongation

**DOI:** 10.1371/journal.pone.0057328

**Published:** 2013-02-21

**Authors:** Pedro Rafael Costa, Marcio Luis Acencio, Ney Lemke

**Affiliations:** Departamento de Física e Biofísica, Instituto de Biociências de Botucatu, UNESP - Univ Estadual Paulista, Botucatu, São Paulo, Brazil; Weizmann Institute of Science, Israel

## Abstract

The transcription process is crucial to life and the enzyme RNA polymerase (RNAP) is the major component of the transcription machinery. The development of single-molecule techniques, such as magnetic and optical tweezers, atomic-force microscopy and single-molecule fluorescence, increased our understanding of the transcription process and complements traditional biochemical studies. Based on these studies, theoretical models have been proposed to explain and predict the kinetics of the RNAP during the polymerization, highlighting the results achieved by models based on the thermodynamic stability of the transcription elongation complex. However, experiments showed that if more than one RNAP initiates from the same promoter, the transcription behavior slightly changes and new phenomenona are observed. We proposed and implemented a theoretical model that considers collisions between RNAPs and predicts their cooperative behavior during multi-round transcription generalizing the Bai *et al.* stochastic sequence-dependent model. In our approach, collisions between elongating enzymes modify their transcription rate values. We performed the simulations in Mathematica® and compared the results of the single and the multiple-molecule transcription with experimental results and other theoretical models. Our multi-round approach can recover several expected behaviors, showing that the transcription process for the studied sequences can be accelerated up to 48% when collisions are allowed: the dwell times on pause sites are reduced as well as the distance that the RNAPs backtracked from backtracking sites.

## Introduction

The first step of the Central Dogma of Molecular Biology concerns the transport of information from the DNA to an RNA molecule. This process, known as *transcription*, must be exquisitely controlled during the development and maintenance of living beings. It can be divided into three phases–*initiation*, *elongation* and *termination*–and is carried out by the RNA polymerase enzyme (RNAP).

The RNAP scans the duplex DNA to find the sites for transcription initiation, known as *promoters*, and bind to them, exposing the DNA template. Once the active site of RNAP is in the correct position, the Transcriptional Elongation Complex (TEC) starts the elongation phase. During this phase, the RNAP polymerizes RNA chains, incorporating a ribonucleoside complementary to the nucleotide present in its active site. After incorporation, the RNAP moves along one nucleotide in the template strand and then restarts the process. When it recognizes the site for termination, the TEC is disassembled and the RNAP releases the transcript and disengages from DNA. Throughout this process, RNAP is able to recruit accessory proteins for several of these activities.

The development of single-molecule techniques, such as magnetic and optical tweezers, atomic-force microscopy and single-molecule fluorescence, increased our understanding of the transcription process, complementing traditional biochemical studies (for a review, see Herbert *et al.* [1]). Kinetics studies showed the occurrence of *transcriptional pauses*. There are two different possible explanations for this phenomenon: interaction between nascent RNA and RNAP or sequence-dependent interactions among RNA, RNAP and DNA. These pauses play an important role in the mechanism of transcriptional regulation. Several works showed that pauses allow the recruitment of regulatory factors and are important for transcriptional termination, among other functions (see the introduction of Herbert *et al.* [2] work for a brief review).

Different approaches have been proposed to model the elongation kinetics. Models based on thermodynamic analysis presented in the pioneering paper of von Hippel and colleagues [3] made possible the prediction of DNA sequence dependent pause sites [4], [5]. Although these models consider the presence of only one transcribing RNAP on the DNA strand, experimental evidence has been gathered to suggest that multiple RNAPs can simultaneously participate in the process. Ribosomal genes, for instance, are highly transcribed and electron micrographs of the process are remarkable for their Christmas-tree-like molecular organization, where the “trunk” of the tree are the DNA strand, and the “branches” are the nascent RNAs in increasing length. It is possible to observe even hundreds of branches, indicating the high RNAP concentration in these genes [6], [7]. There are two different views on the impact of this multi-round transcription. The first view is that the leading RNAP blocks the advancement of the trailing ones at pause sites, inducing traffic jams [8]. The other one is that the trailing RNAP prevents backtracking and pushes the leading RNAP out of pause sites [9]. The later view corroborates Epshtein and Nudler [10] work, that compared the single and the multi-round transcriptions, showing that in the presence of multiple active enzymes a larger number of RNAP can leave an arrest site and continue the elongation. In another work, Epshtein *et al.* [11] showed that efficient transcription through proteins roadblocks depends on multiple rounds of initiation. They also conclude that the trailing RNAP molecules can rescue roadblocked complexes in vivo by “pushing” them forward.

Klumpp [12] presented a model to study the pauses and backtracking under dense traffic conditions. His approach distributes the pauses and the backtracking sites along the DNA template according to a density parameter, neglecting the dependence of the local DNA sequence on the transcription rates. He drew the conclusion that the pauses are suppressed due to the fact that the leading polymerase backtracking is restricted in the presence of a trailing RNAP. Rajala and colleagues [13] concluded, using a delayed stochastic model, that the rate of occurrence and duration of the pauses affect the “microbursting” of RNA production. According to them, these bursts occur when two or more RNAPs complete the transcription of the same gene within a shorter interval than the expected minimum interval between consecutive initiations. A long-pause site can drastically alter the distribution of bursts without changing mean expression levels. Tripathi and Chowdhury [14] proposed a sequence-independent model that also considers collisions and traffic jams and investigated the impact of RNAP interactions on the fluctuations in the synthesis of RNA.

In this current work, we propose a multiple-round elongation approach (*MRA*) that identifies and solves collisions between the RNAP molecules. This approach is based on the thermodynamic sequence-dependent model developed by Bai *et al.* [4], hereafter referred as *Bai model*. A brief recapitulation of this model is presented on the Methods section. The *Bai model* was reimplemented using updated parameters and referred as single-round transcription approach, *SRA*.

## Methods

### A brief recapitulation of Bai's thermodynamic sequence-dependent model

**Figure 1:**
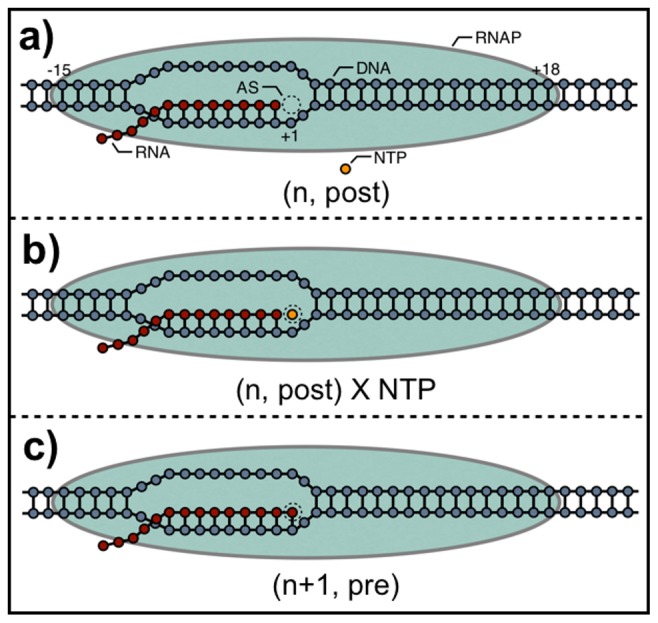
Representation of transcription elongation complex (TEC): The TEC is given by the RNA length, 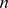, and by the relative position of the Active Site, *AS*, to the 3′ end of the RNA. a) Post-translocated state: active site free. The TEC structure is given by an RNA-DNA hybrid 8 bp long and by a DNA bubble with 12 bp. b) Post-translocated state, incorporation phase: active site just occupied, NTP being incorporated to RNA. c) Pre-translocated state: active site occupied and the NTP incorporated in RNA. Here, the RNA-DNA hybrid is 9 bp long and the TEC will move one nucleotide forward, returning to a).

The *Bai model* has been chosen due to its simplicity and consistency with experimental results. This model is based on the thermal ratchet model for molecular motors and considers the elongation as a three-step reaction:
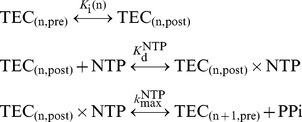
(1)
**Eq. 1** includes the TEC translocation, the nucleotide specific (NTP) binding and the chemical catalysis. The pyrophosphate anion is abbreviated as PPi. The 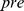 and 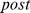 represent the TEC configuration and 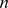 is the transcript length. **Figure 1** depicts the TEC structure. 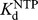 and 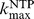 were experimentally established and depend on the nucleotide to be incorporated. Their values are shown on **Table 1**. 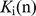 is given by
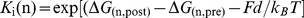
(2)where 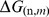 is the standard Gibbs free energy for the TEC conformation and 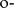 is an external force applied to RNAP, with 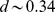 nm being the distance between adjacent nucleotides in the DNA strand [15]. 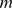 gives us the position of the active site of RNAP on the template. It is equivalent to use 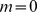 or 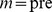 and 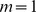 or 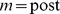.

**Table 1:** Parameters Values.

	ATP	UTP	GTP	CTP
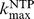 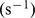	50 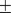 6	18 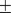 1	36 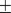 5	33 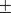 6
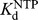 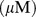	38 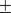 7	24 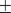 4	62 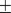 18	7 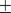 4

Parameters values for **Eq. 3**. These NTP-dependent parameters were experimentally determined by Bai *et al.* [15].

Using the Michaelis-Menten kinetics, we can obtain the overall rate for each ribonucleoside incorporation [16]:
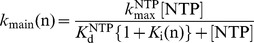
(3)Finally, there is also a backtracking/forwardtracking rate given by
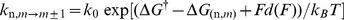
(4)where 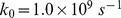 is a pre-factor constant and 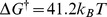 is the energy barrier for this phenomenon.

#### The standard Gibbs free energy, (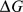), for the TEC

The 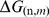 for the TEC, first described by Yager and von Hippel [3], is a measure of its stability, and is given by the sum of the isolated components:
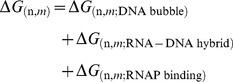
(5)


The first term is the free energy due to the break of the hydrogen bonds between complementary nucleotides on the double-strand DNA and the second term is the free energy due to the formation of the RNA-DNA hybrid duplex. They are clearly sequence-dependent and we use the nearest-neighbor model for both [17]. We used SantaLucia *et al.* [18] values for the DNA-DNA energy and Sugimoto *et al.* [19] values for the RNA-DNA hybrid energy. The third term represents the interactions between the RNAP and the nucleic acids, considered sequence-independent and, for simplification, as zero. For further details and considerations on these calculation, see Bai *et al.* [4].

### The multiple-round approach

**Figure 2:**
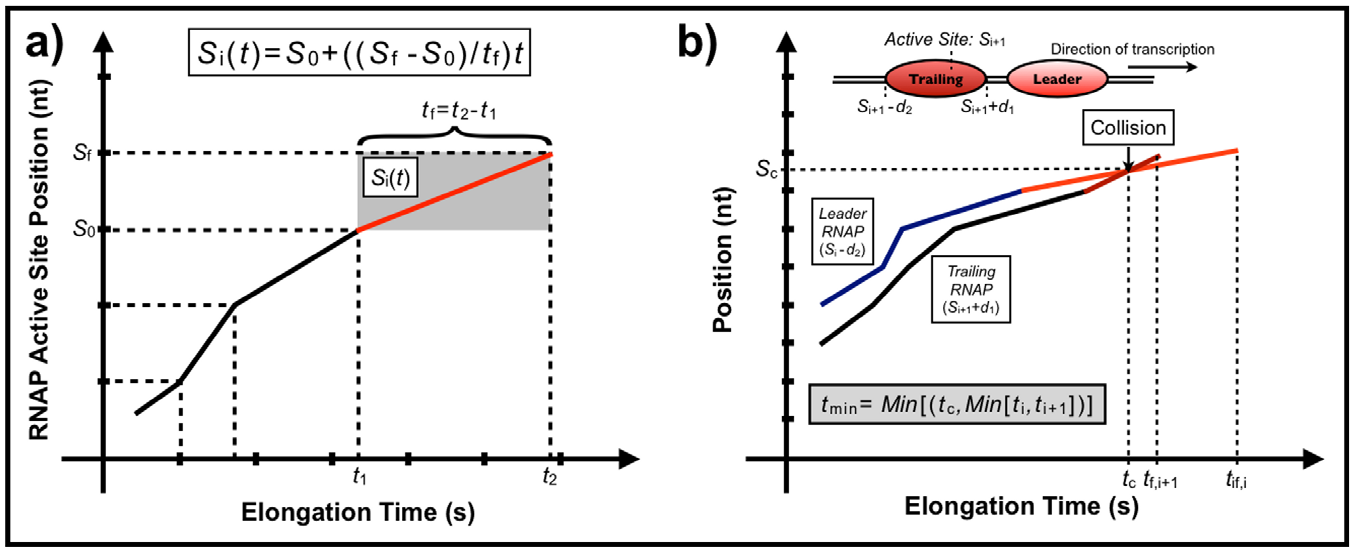
Representation of time evolution of RNAP: The red lines represent the last simulated step. a) The time evolution for a single molecule. We consider that the movement is uniform between each step. The velocity is assigned by the Gillespie algorithm. b) The method used to solve collisions. In blue we show the leader RNAP and in black the trailing RNAP. 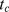 represents the required time for the collision. 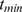 is the time for the first event among the following possibilities: nucleotide inclusion by trailing RNAP, nucleotide inclusion by leading RNAP or collision.

The multiple-round approach extends the Gillespie algorithm [20] to consider collisions between RNAP molecules. Essentially, the algorithm is summarized below:

1. We generate a simulation for each RNAP on the DNA strand. If the RNAP is in the normal elongation, we calculate the reaction rates using **Eq. 3** and **Eq. 4**. If the RNAP is backtracking, we use **Eq. 4** to determine the rates for the backtracking/forwardtracking reaction. For both cases, we set 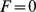. The simulations will return the required time for each RNAP complete its movement.
2. A linear approximation was used to determine the time-dependent position equation for each enzyme 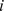, 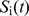. **Figure 2a** shows a diagram to illustrate the method.
3. The algorithm determines the required time for collision between each subsequent pair of enzymes by solving 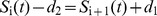, where 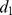 and 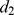 represent the distances between the active site of the RNAP to its ends. A sketch of a collision case between two RNAPs is presented in **Figure 2b**. The algorithm determines which event occurs first: if an RNAP completes its movement or if we have a collision.
4. All the RNAPs are repositioned: using the time of the event determined in item 3 in their respective 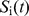, we determine where each molecule is, and a new position equation for all of them. If the event was a collision, we solve the collision according to its type, what will change the position equation for both molecules (see below) and go back to item 3. Otherwise, we go back to item 1.

**Figure 3:**
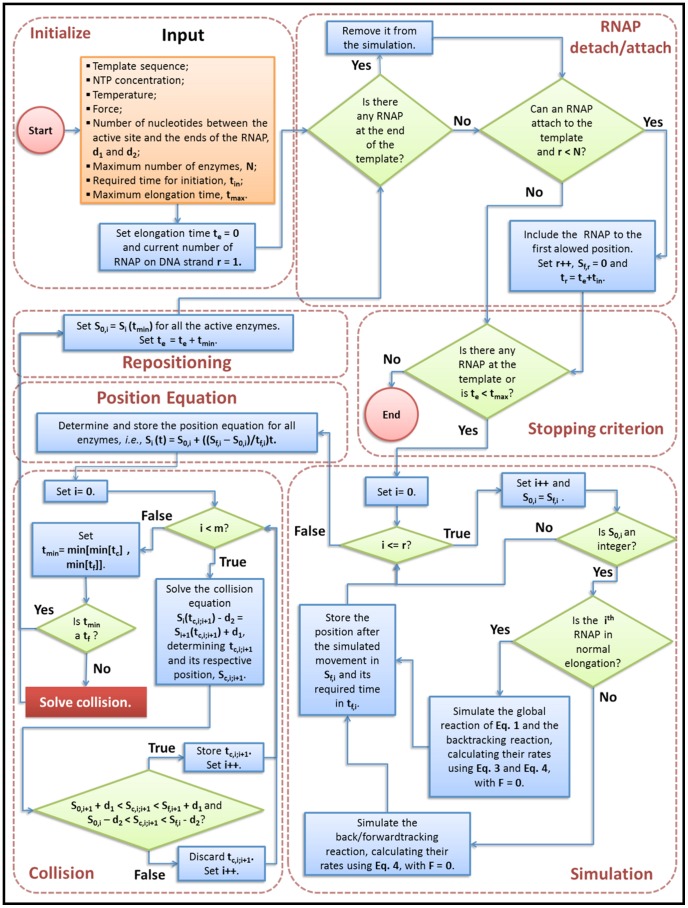
Diagram representing the overall algorithm: The subroutine “Solving collision” is described in **Figure 4**.

**Figure 4:**
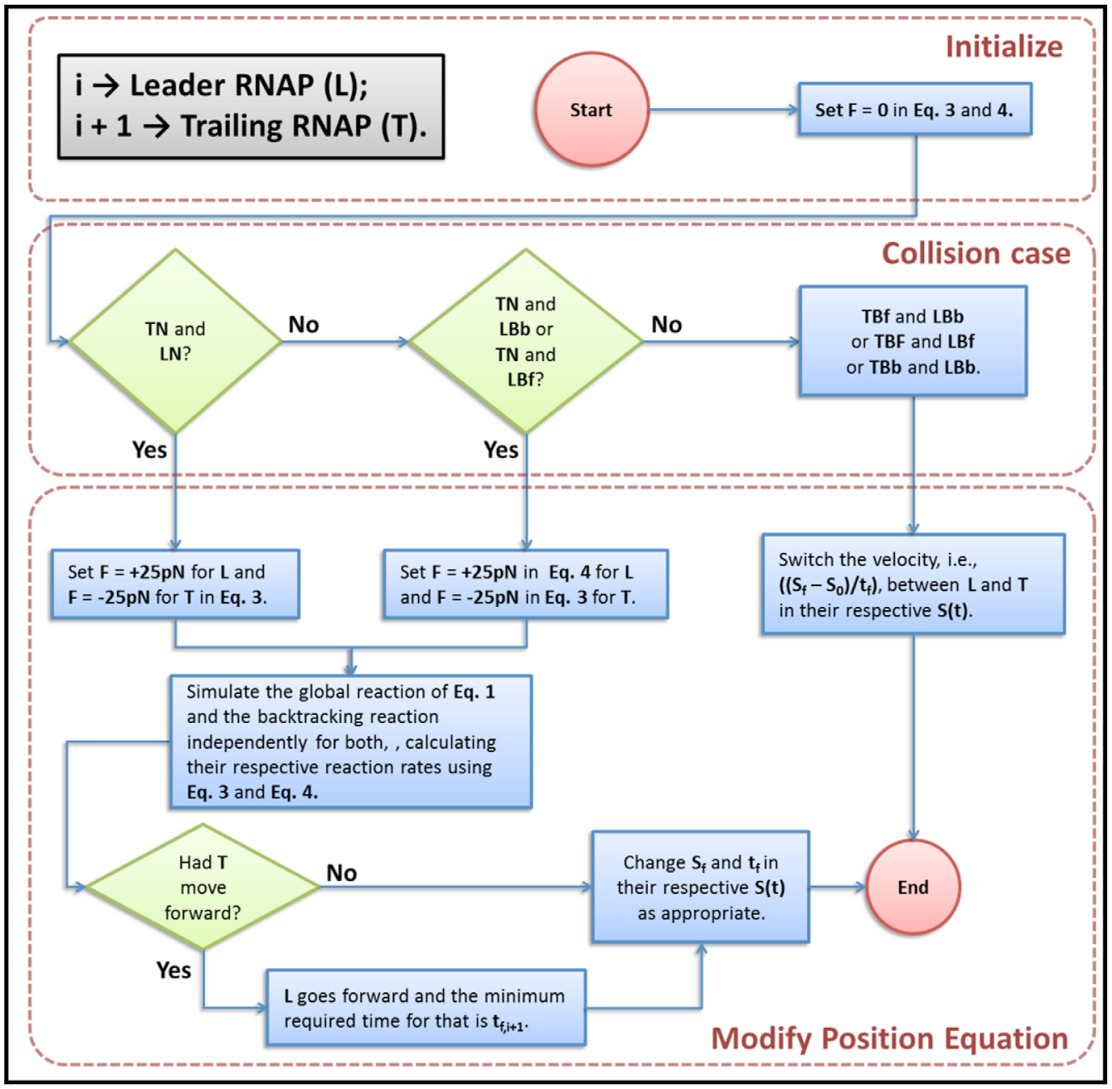
Diagram representing the subroutine to solve collisions: **TN** mean trailing RNAP in normal elongation. **LN** means leading RNAP in normal elongation. **LBb** means leading RNAP in backtracking backwards movement. **LBf** means leading RNAP in backtracking forwards movement. **TBb** means trailing RNAP in backtracking backwards movement. **TBf** means trailing RNAP in backtracking forwards movement.

During the simulation, both the trailing RNAP molecule (*T*) and the leader one (*L*) can be in normal elongation, or in backtracking, moving backwards or forwards. Due to this, the collision between the RNAP molecules can be categorized in six different types. Basically, if *T* is in normal elongation, it applies 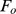 on *L*. As the result, *L* applies 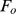 on *T* in the opposite direction. If *L* is backtracking and after the collision *T* moves forwards, *T* pushes *L*. If they both are backtracking, we treat the collision as being perfectly elastic. In **Figures 3** and **4** we present diagrams depicting the algorithm. **Figure 4** details the subroutine to solve collisions.

### Simulations

We used Monte Carlo simulation with the Gillespie algorithm (first reaction method) [20], using the SSA (Stochastic Simulation Algorithm) package [21] for Mathematica®. A complementary package was developed with functions to implement the single and multiple-round approaches. We performed 4800 independent simulations of the kinetics up to 10 RNAP molecules on the same DNA strand and we set the required time for initiation to 0.5 s. We also simulate different cases varying the force parameter, 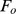, from 6.25 pN to 100 pN. The sequences considered correspond to the deletions D104, D111, D112, D123, D167 and D387 of the early genetic region of the bacteriophage T7 [22] ([NTP]: 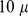
M for pause sites and 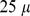
M for the simulated gel) and seq10 to seq13 from Tadigotla *et al.* [5] ([NTP]: 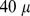
M for seq10, 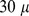
M for the others). All the reactions were simulated at 24
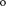
C.

### Pause Criteria

Here, we used the same pause criteria proposed by Bai and Wang in [23]. A pause site is defined by
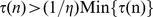
(6)where 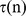 is the dwell time for each the template position 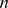 and 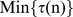 is the shortest 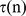 on the template at a given NTP concentration. The parameter 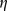 is tunned to optimize the pause predictions for all the studied sequences. As Tadigotla *et al.* in [5], we choose to minimize the proportion of incorrect to correct predictions (false positives plus false negatives divided by true positives). Here, we use the third quartile values for 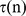. Although the 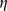 value for *Bai model* was 0.05, here we set 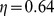 for *SRA*, and 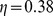 for *MRA*. Expressing them as a dwell time threshold, we have about 0.8 s for *SRA* and 1.4 s for *MRA*.

## Results and Discussion

We developed an extension of the *Bai model* for RNAP transcription kinetics that considers interactions among multiple RNAPs. We compared the model with existing experimental data and discussed the possible biological implications emerging from these interactions. Initially, we set 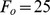 pN. This value was used because it is the average stalling force of RNAP [24].

**Figure 5:**
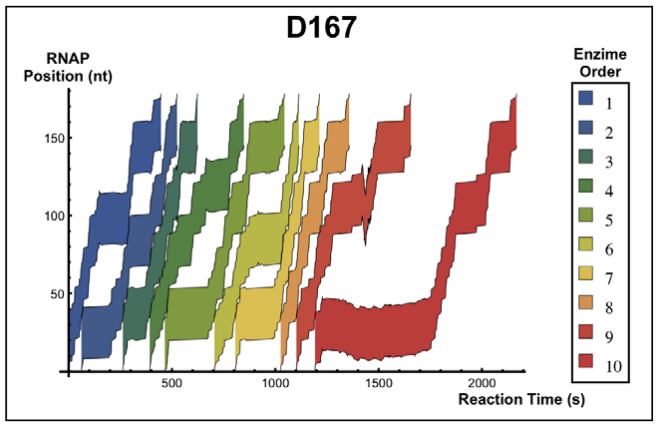
RNAP position in function of reaction time during a simulation of the *MRA*: We can see the kinetic behavior of the RNAP during the *MRA* for sequence D167: the evolution in time of the position of the enzyme in the DNA strand. Each region represents the space occupied by the enzyme during transcription, and the colors indicate the binding order to the DNA strand. Note that there is no overlap between the regions. Points where the regions touch each other indicate the occurrence of a collision between the molecules. The colored regions represent the space occupied by the enzymes during the reaction. Sites where molecules take longer dwell time to continue transcription and backtracking sites are easy to identify. The collisions between the molecules usually occur at the pause candidate sites.

**Figure 5** shows a result obtained during a simulation run of the *MRA*, i.e., the kinetic of the transcribing RNAPs in the DNA strand. As we can observe the dynamics is intermittent: random walk-like movements coexist with strongly directed progressive motion. The regions where the random walks prevails are considered strong pause sites. The random walk movement is usually interrupted after a collision between two RNAPs. From a qualitative point of view, we can see that collisions can increase the pace of transcription. Moreover, we can easily see that there are regions on templates more prone to be pause sites indicating that this behavior is sequence-dependent. Experimentally, these regions can be detected on transcription gels. In the remaining article, we will explore this qualitative picture in further details.

**Figure 6:**
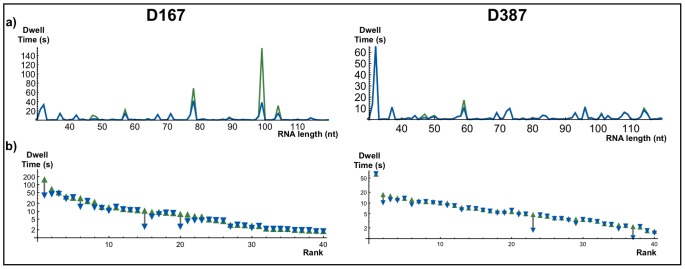
Elongation kinetics for sequences D167 and D387: a) Dwell times between nucleotide incorporation by the RNA strand length. Green: *SRA*; Blue: *MRA*. The lines are almost overlapped: differences between them arise only in sites with larger dwell times. b) Forty larger values of dwell time for *SRA* (upward triangle, green) and the value for the correspondent site in *MRA* (downward triangle, blue). The graph is on a logarithmic scale.

We compared the biological consequences of collisions shown in **Figure 6** for sequences D167 and D387. **Figure 6a** shows the third quartile of dwell time (TQDT) distribution for nucleotide inclusion in function of RNA length. In **Figure 6b** we present the ranked TQDT obtained on single round approach. We compared these values with the respective TQDT obtained in the same RNA length for multiple round approach. We restricted ourselves to the 40 largest dwell time values because these dwell times are more susceptible to collision effects. In general longer TQDTs are more affected by collisions, but the pause position on the sequence also plays a role. For example, in D387 we can clearly observe a strong pause in the beginning of the sequence that remains unaltered even considering collisions. This can be explained since the active enzyme does not allow the attachment of another RNAP to the DNA strand. Another example of the effect of pause position can be seen at the positions 60 and 70. At position 70, the inclusion time is not affected while the time for position 60 is almost halved. This behavior can be understood by observing that at the position 70, the TQDT is shorter than the typical time that a RNAP takes to move from position 60 to 70. Nevertheless, the TQDTs are always reduced when we consider multiple RNAPs. Despite the strong sequence dependence of this effect, the collisions significantly accelerate the passage of RNAPs by the pause sites. The gain in performance occurs mainly on the top 5% dwell times: without collisions they cost 44% of the required time for elongation. When we consider collisions this value is reduced to 27%.

**Figure 7:**
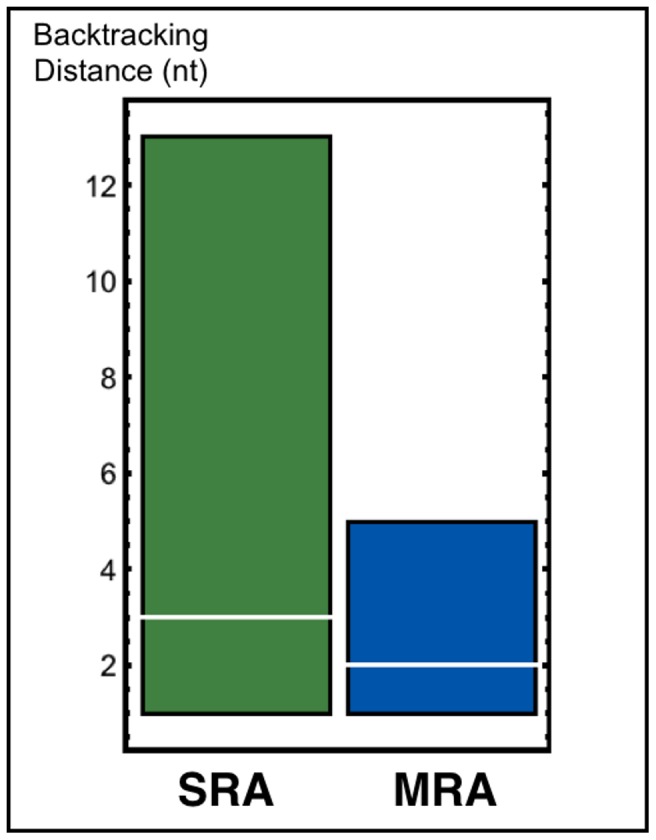
Distribution of the distances covered by backtracked RNAPs from the backtracking site: The lower and upper limits of the boxes correspond respectively to the first and third quartiles. The median is highlighted in white.

**Figure 8:**
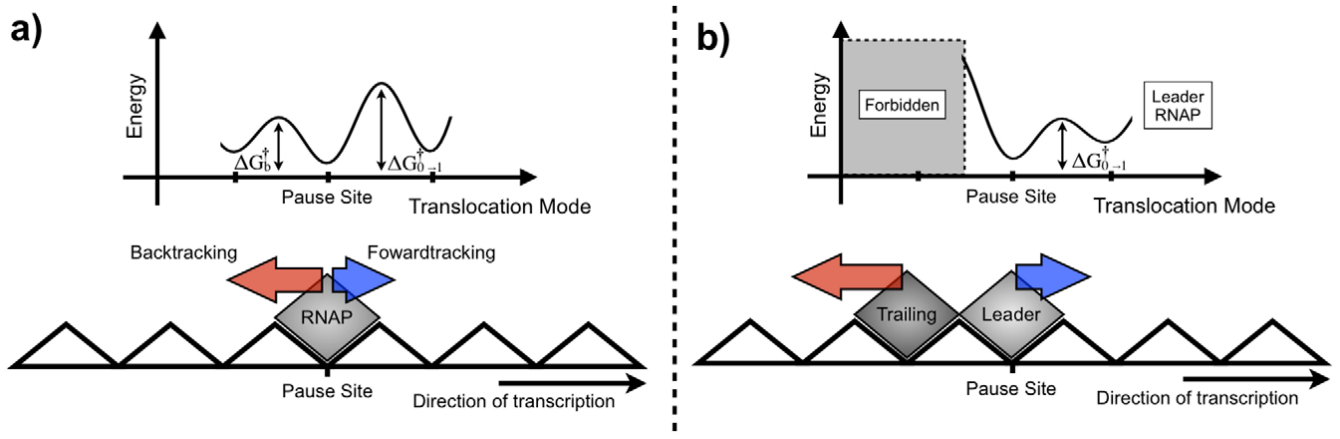
Schematic view of the roadblock effect: a) At a pause site the backtracking probability is higher than the probability of normal elongation. b) The trailing RNAP acts a roadblock preventing the backward movement of the leader RNAP.

The suppression of backtracking, noticed by Epshtein *et al.* [11] and reproduced by Klumpp's model [12], is also observed in our model. The distribution of the distances covered by backtracked RNAPs from the backtracking site during all the simulations can be seen in **Figure 7**. These distributions have approximately the same median, but it is possible to notice a 2.6-fold reduction in the third quartile of backtracking distance for multiple RNAPs in comparison to single RNAP. Trailing RNAPs are physical barriers that prevent backtracking and the collisions between trailing and leader RNAPs induce a forward movement (see **Figure 8**). Among the possible biological consequences, we can highlight the reduction of transcriptional proofreading efficiency as suggested by the theoretical study of Sahoo and Klumpp [25].

**Figure 9:**
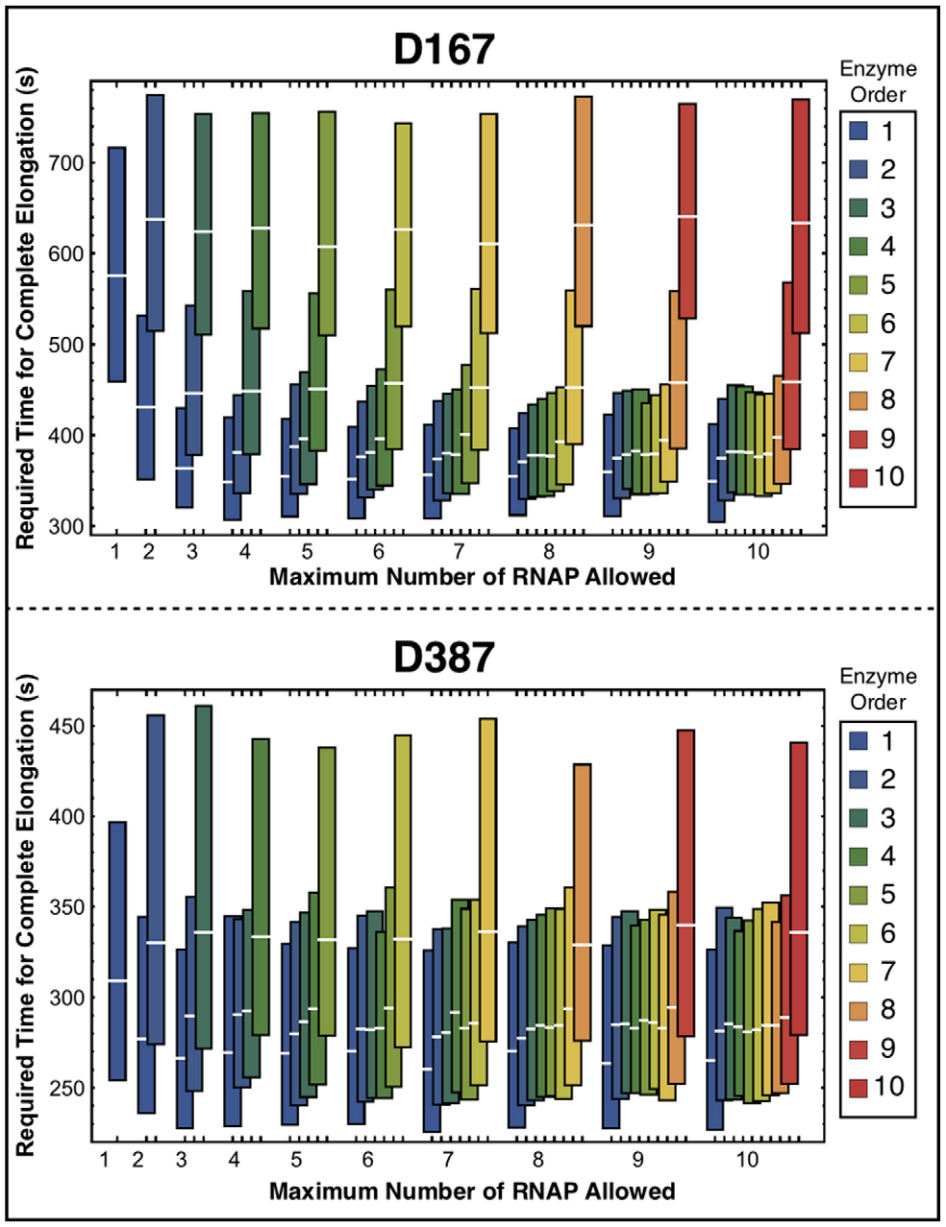
Required time for complete elongation for all RNAPs by the maximum number of RNAP allowed on the template: Distribution of the 4800 required times to complete transcription of the template by the maximum number of RNAP allowed. The upper and lower limits of each bar indicates respectively the first and third quartiles of these distributions, with a median highlighted in white. The colors represent the RNAP binding order.

To evaluate the overall effects of multi-round transcription, we determined the distribution of required times for a complete elongation for each enzyme in each DNA template. These results are presented in **Figure 9**. The distribution of transcription times weakly depends on the RNAP order except for the last enzyme. The last RNAP is not accelerated by collisions and is restrained to move forward by other enzymes. This explains why this molecule takes longer time to transcribe than the RNAP in the *SRA* simulation. This behavior persists for all studied sequences.

**Figure 10:**
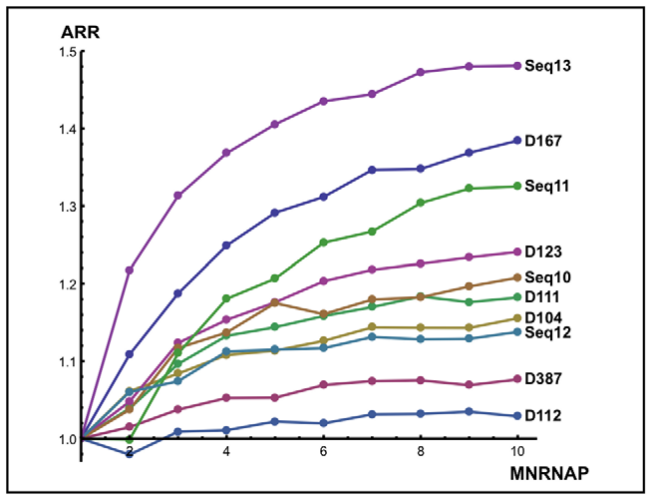
Relative transcription efficiency, 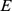
 **:** Each color represents a sequence, as indicated at the end of the respective curve. The 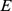 values are shown as a function of the maximum number of enzymes 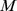 allowed during the simulation. Note that the asymptotic values of curves are sequence dependent.

To characterize the efficiency of the multi-round transcription, we defined the average transcription time by:
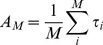
(7)where 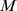 is the maximum number of molecules on the template, 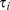 is the median of the distribution of required times for the completion of transcription for 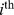 enzyme and the relative transcription efficiency by:
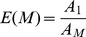
(8)The values for 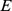 are shown in **Figure 10**. We can see that 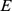 is an asymptotic increasing function of 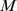 where the asymptote is strongly sequence dependent, clearly indicating the cooperative nature of RNAP interactions. For example, the asymptotic 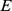 for sequence D112 is only 1.03, while the asymptotic 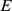 for Seq13 is 1.48. The finite length of the template imposes a definite limit for 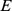, since there is a finite number of simultaneously transcribing RNAPs. Furthermore, the number, intensity and position of the pauses are also limiting factors: for example, intense pauses at the beginning of the sequence, such as in the D387, restrict the presence of several active RNAPs on the template.

**Figure 11:**
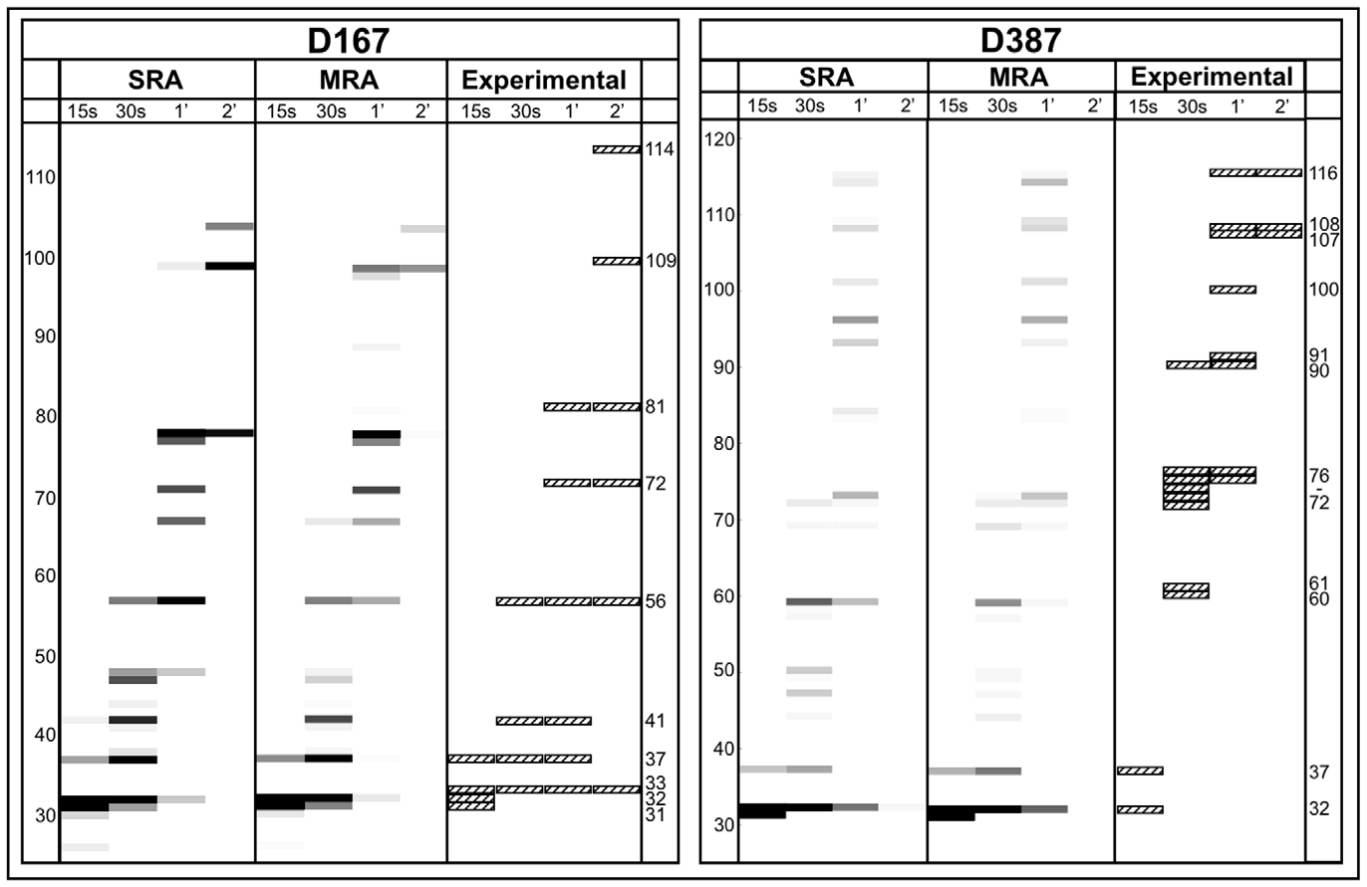
Comparison among the simulated transcription gels in different times: a) Template D167. b) Template D387. Each column represents the reaction time for that simulation. The intensity of the bands is proportional to the amount of produced transcripts of that length in the allowed time. The experimental results shown here are graphical reproductions of the gels presented in the experimental work of Bai and Wang [23]. The experimentally observed bands are indicated with their respective transcript size. Some false positives present in the *SRA* appear less intense in the *MRA*. For the single-round and the multi-round elongation: some bands appear earlier in *MRA* than in *SRA* and the predicted intensities are different for most bands.

**Figure 11** shows the comparison of the predicted transcription gels by *MRA* and *SRA* in particular times with the experimental results [23]. It is important to stress that the multiple round approach does not represent these experimental conditions, since in this case we have a single transcribing RNAP. To quantitatively measure the relative prediction performance of the different models, we used a quality metric originally proposed by Tadiglota *et al.* [5]: first, pause sites are grouped in clusters in which pauses within 3 bp are considered as a single cluster, and then the number of detected (True Positives, 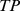) and undetected (False Positives, 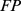) experimental clusters are counted. Finally, these factors are incorporated in a quality metric defined as:
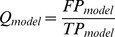
(9)


**Figure 12:**
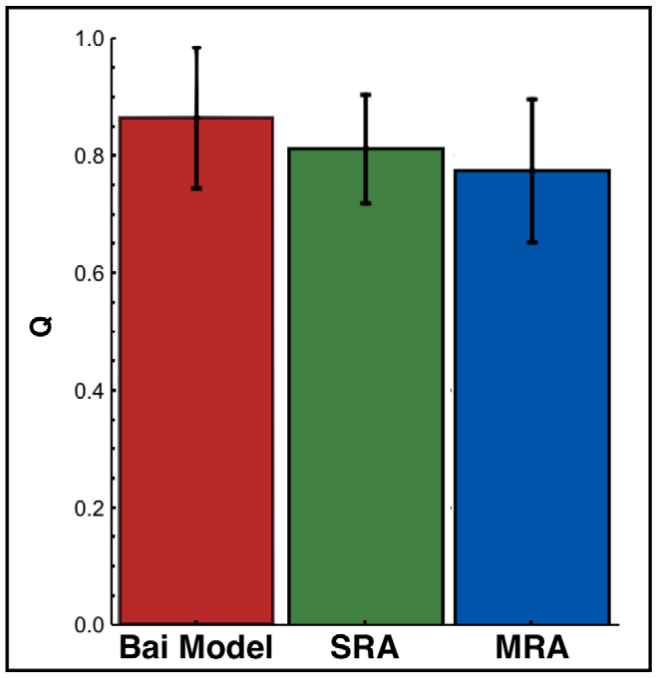
Relation between the incorrect and correct prediction, 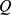: The error bars represent the standard deviation obtained by a bootstrap technique. In this case, we evaluated the impact of the finite number of studied sequences on 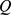.

**Figure 12** shows 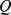 for the different models: *Bai model*, *SRA*, and *MRA*. The use of updated parameters improved pause detection compared to *Bai model*. The RNAP collisions do not impact significantly on the pauses position, but the 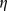 parameter, that is strongly related to transcription efficiency, varies from 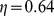 (*SRA*) to 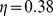 (MRA). The large error bars are due to the small number of experimental data.

**Figure 13:**
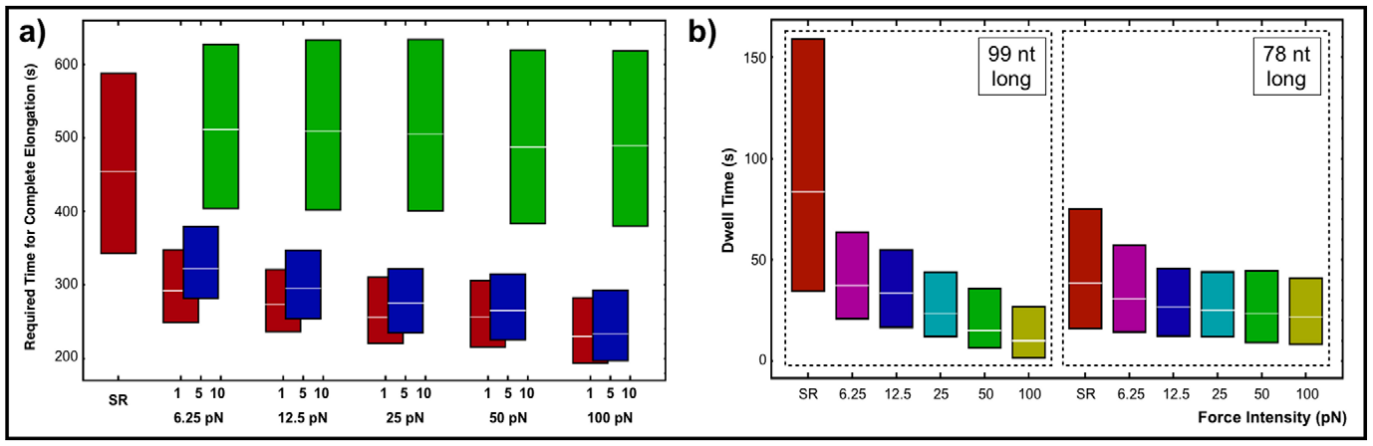
Force influence on multiple-round approach: a) Force influence on the required time for complete elongation. Red bars represent the first RNAP, blue bars represent the fifth RNAP and the green bar represents the tenth RNAP. b) Force influence on the dwell times for two representative nucleotide inclusions.

On **Figure 13a** we compared the required time for complete elongation for different values of the force parameter for sequence D167. For the first and the fifth RNAP we observe a decrease on the total required elongation time with increasing force. For the last RNAP the effect is negligible. We also present the result for a single RNAP for comparison purposes. The effect of the pushing force is small when compared to the roadblock effect (see **Figure 8**). On **Figure 13b** we show that the force influence is strongly sequence dependent by comparing dwell times for two different transcripts lengths. The pause site located at the position 99 with a longer dwell time than the one located at position 78 suffers a stronger influence of the cooperative RNAP behavior.

In summary, our results are in accordance with the literature, showing that the multi-round transcription increases the overall transcription rate by reducing pauses duration and by suppressing RNAP backtracking. The low 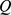 parameter indicates that some key ingredients to describe this scenario are still missing. An interesting possibility is to propose a more detailed model for the nascent transcript considering different possible RNA structures and their influence on the pace of transcription. Although we can speculate that pauses can be used as regulation factors by the cell, the cooperative behavior of RNAPs reduces the variability of the required time for complete elongation. Our model could be extended to deal with other experimental conditions such as the cooperative action of RNAPs in overcoming the nucleosomal barrier [26].
